# Noninvasive Novel Transdermal Drug Delivery System for Deep Drug Permeability

**DOI:** 10.34133/research.0504

**Published:** 2024-10-14

**Authors:** Huijie Han, Hélder A. Santos

**Affiliations:** ^1^ Department of Orthopaedics, Shanghai Key Laboratory for Prevention and Treatment of Bone and Joint Diseases, Shanghai Institute of Traumatology and Orthopaedics, Ruijin Hospital, Shanghai Jiao Tong University School of Medicine, Shanghai 200025, China.; ^2^ Department of Biomaterials and Biomedical Technology, The Personalized Medicine Research Institute (PRECISION), University Medical Center Groningen (UMCG), University of Groningen Ant. Deusinglaan 1, Groningen 9713 AV, The Netherlands.; ^3^ Drug Research Program, Division of Pharmaceutical Chemistry and Technology, Faculty of Pharmacy, University of Helsinki, Helsinki FI-00014, Finland.

Noninvasive drug delivery systems are well applied due to their safety, convenience, and patient compliance [1]. However, drug permeability in traditional noninvasive drug delivery systems is limited, and it is difficult to regulate the administration time and dosage [2]. The depth of drug permeability is mainly impeded by geometry obstruction and diffusional resistance of the stratum corneum, which consists of 10 to 30 layers of keratinized corneocytes embedded in an extracellular lipid matrix. It is a great challenge to overcome stratum corneum to enhance drug permeation across skin [3]. The latest publication in *Advanced Materials* from the teams of Cai, Cui, and Bai introduced a new type of nanobubble ultrasonic coupling hydrogel, which is built by dynamic Schiff base cross-linking between nanobubbles and aminated hyaluronic acid (HA) [4]. This ultrasound coupling hydrogel opens new insights in noninvasive drug delivery because it efficiently overcomes the skin barrier and achieves precise spatiotemporal drug delivery by ultrasound-amplified cavitation effect.

Transdermal drug delivery is one of the most common drug delivery systems and plays the leading role in noninvasive drug delivery [5]. It not only is noninvasive and convenient but also avoids the first-pass effect and increases drug bioavailability [5]. Currently, there are several chemical, physical, and biological methods for transdermal drug delivery [6]. Although there is some progress in chemical and biological methods, such as chemical absorption enhancers and biological mucosal adhesives, the ideal drug permeability accelerator is still in exploration [7,8] since drug absorption enhancers may damage epithelium mucosae, while mucosal adhesives show limited drug permeability efficiency [9]. As for physical transdermal drug delivery, the most common method is to destroy the skin barrier through subcutaneous injections, such as microneedles, but such a strategy has not achieved complete noninvasion and efficient drug permeability [10], and the drug permeability inefficiency is largely dependent on the length of the microneedles [11]. The drug delivery stated in the abovementioned approach has an important effect on drug toxicity, pharmacodynamics, and immunity. There is still a long way to go in optimizing the physical transdermal drug delivery for better drug permeability efficiency.

As the mechanical wave, ultrasound has been widely used in rehabilitation medicine, especially in soft tissue repair, nerve regeneration, and scar hyperplasia prevention [12–14]. Ultrasound greatly promotes hydrophilic and hydrophobic drug permeability, including macromolecules like insulin [15], toxins from enterotoxigenic *Escherichia coli* [16], DNA [17], small interfering RNA [18], bovine serum albumin [19], etc. Furthermore, ultrasound enables constant or specific drug release in certain sites with precise drug dosages. However, ultrasound cannot be effectively transmitted in the air, so medical coupling agents act as a medium to reduce acoustic resistance and improve image quality [20]. Traditional coupling agents are usually liquid conductive media with low nanobubble content and lack mechanically responsive groups. Thus, the cavitation effects of drugs cannot be well enhanced, and the skin stratum corneum and basement membrane barriers cannot be overcome, leading to poor drug permeability efficiency [21–23]. Therefore, there is an urgent need for a new type of ultrasound coupling agent to improve drug permeation and release.

Recently, the teams of Cai, Cui, and Bai reported a new type of nanobubble ultrasound coupling agent in *Advanced Materials* [4]. This type of coupling agent integrates ultrasonic real-time imaging and noninvasive deep drug permeation by overcoming double barriers of the skin. To achieve this, the ultrasound coupling agent crosslinked perfluorohexane-modified celecoxib-loaded poly(lactic-co-glycolic) (Cel-PLGA) acid nanobubbles with aldehyde HA and aminated HA hydrogel by Schiff base reaction (Figure). Celecoxib was successfully permeated into the dermis layers with 2.5-fold higher permeability efficiency. The long-term efficacy of the coupling agent was evaluated in the tendon adhesion rat model and demonstrated that celecoxib was successfully delivered in the gap between skin and tendon, reducing the adhesion degree after tendon injury. Biosafety and biocompatibility were evaluated in vitro by live-dead staining, skeletal staining, and cell viability of fibroblasts (F208), and the result indicated great biocompatibility of coupling agents without fibroblast toxicity in 5 d. In summary, unlike traditional coupling agents, the new type of coupling agent can efficiently transfer drugs through the double barrier of the skin by amplifying the cavitation effect of ultrasound, with a penetration depth of up to 728 μm in a noninvasive way.

**Figure.**
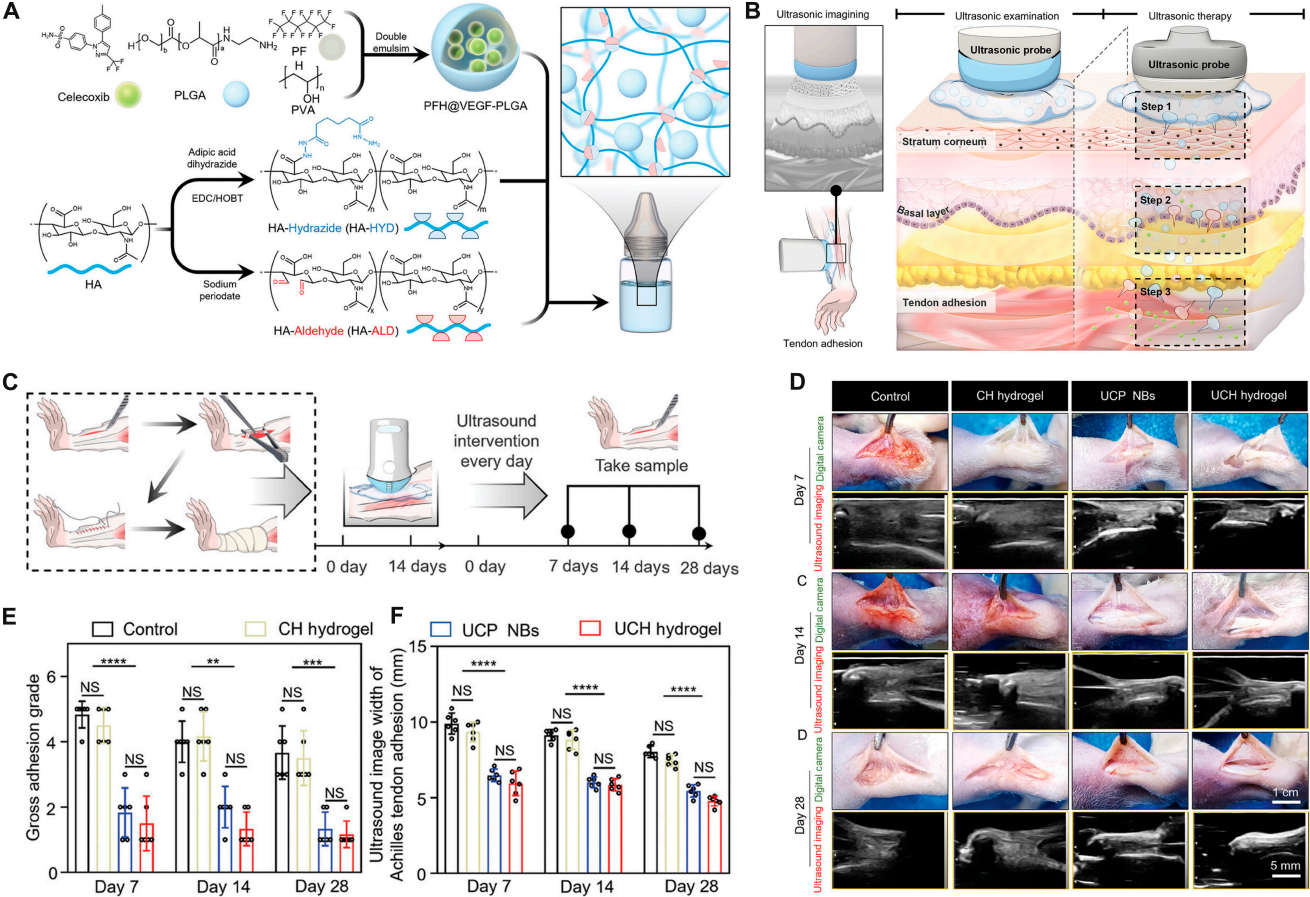
Schematic diagram and therapeutic effect of ultrasound nanobubble coupling agent for effective noninvasive deep-layer drug delivery. (A) Fabrication process of Cel-PLGA nanobubbles (Cel NBs). (B) Fabrication of Ultrasound@Cel NBs@HA. (C) Tendon adhesion model in a rat with a schematic illustration of the surgical procedure. (D) Macroscopic and ultrasound images of rat Achilles tendon adhesion in different treatment groups at 7, 14, and 28 d. (E) Macroscopic and ultrasound scoring of Achilles tendon adhesion in different treatment groups at 7, 14, and 28 d. Reprinted with permission from [4]. Copyright 2024, Wiley-VCH GmbH.

Low-intensity ultrasound-based transdermal drug delivery has its unique advantages over traditional drug delivery due to its noninvasive and avoidance of the first-pass effect [24]. Although nanobubble ultrasound coupling agents are found to enable noninvasive transdermal drug delivery and real-time imaging of targeted lesions in in vivo studies [25], their clinical applications are still in an early stage. In 2024, a clinical trial optimized postoperative pain control after laparoscopic colorectal surgery by supplementing ultrasound-guided transdermal Fentanyl patch (https://clinicaltrials.gov/), which indicated the great potential of ultrasound nanobubbles in small-molecule drug delivery. However, challenges still exist in the clinical application and translation. For example, drug permeating depth is still limited, especially in obesity with thick subcutaneous and cancer patients with deep tumor lesions [26]. In addition, because ultrasound-based transdermal drug delivery is energy-driven by active devices, portable and wearable ultrasound patches are usually necessary for wider applications. To address these problems, nanobubble sizes [27], concentrations [28], compositions [29], and ultrasound density [30] are adjusted to optimize drug permeation, while small-sized low-frequency transducers, like class V flextensional transducer, are developed to produce ultrasound in portable approach [31]. Therefore, ultrasound-based efficient, safe, and precise drug delivery, from small-molecule to macro-biomolecule drugs, provides more opportunities in therapeutic and diagnostic areas [32].
